# Visual Function Assessment in Geographic Atrophy: A Review

**DOI:** 10.1111/ceo.70037

**Published:** 2025-12-12

**Authors:** Ye Li, Lauren N. Ayton, Adrian T. Fung

**Affiliations:** ^1^ Department of Ophthalmology Royal Brisbane and Women's Hospital Brisbane Australia; ^2^ Department of Optometry and Vision Sciences University of Melbourne Melbourne Australia; ^3^ Department of Surgery (Ophthalmology) University of Melbourne Melbourne Australia; ^4^ Centre for Eye Research Australia, Royal Victorian Eye and Ear Hospital East Melbourne Australia; ^5^ Save Sight Institute, Specialty of Clinical Ophthalmology and Eye Health, The University of Sydney Sydney Australia; ^6^ Department of Ophthalmology Westmead Hospital Sydney Australia; ^7^ Department of Ophthalmology, Faculty of Medicine and Health Sciences Macquarie University Sydney Australia; ^8^ Zhongshan Ophthalmic Centre, Sun Yat‐sen University Guangzhou China

**Keywords:** functional vision, geographic atrophy, vision function

## Abstract

Geographic atrophy (GA) causes significant vision impairment and reduction in vision‐related quality of life. Fundus autofluorescence (FAF) is the gold standard of structural assessment of GA but is a surrogate marker for vision loss, which can be assessed by tests of visual function and functional vision. Best corrected visual acuity (BCVA), the most commonly used visual function test in ophthalmology, is a poor metric for assessing GA progression. This is because GA usually only affects the fovea in its late stage, grows slowly, and spared areas of retina may not ‘fit’ larger reading chart letters, confounding measurements. For this reason, tests of visual function have been developed, including low luminance visual acuity (LLVA), reading speed, contrast sensitivity, microperimetry, flicker perimetry, and dark adaptation. Functional vision measures are approximated through patient‐reported outcomes using various questionnaires. This review explores the strength of association between FAF and tests of visual function in patients with GA. A range of targeted, prespecified endpoints of visual function testing should be included in future clinical trials for treatments of GA, focusing on GA lesion phenotypes that are known to progress rapidly in order to maximise the likelihood of identifying positive results. This is critical in jurisdictions where proof of functional benefit is required for regulatory approval of treatments for GA.

## 1 Introduction

Age‐related macular degeneration (AMD) is the most common cause of visual impairment in patients over the age of 55 in the developed world, with an estimated global prevalence of 8.69% [1]. Geographic atrophy (GA) is a form of advanced AMD that occurs as a result of outer retinal and retinal pigment epithelium (RPE) atrophy, which can progressively and severely compromise central vision. Evaluation of GA extent and progression can be performed by tests of structure and visual function, with the impact of these on activities of daily living (ADLs) seen in tests of functional vision.

Structural assessment of GA is well established and involves multimodal imaging, including fundus autofluorescence (FAF), near‐infrared reflectance, near‐infrared autofluorescence, fluorescein angiography, optical coherence tomography (OCT), OCT angiography, and adaptive optics [2]. Of these, the current gold standard is FAF [3]. However, structural assessment is only a surrogate measure for visual function and often correlates poorly with VA, particularly in cases of extrafoveal GA [4]. Structural tests alone are insufficient in some jurisdictions for the approval of treatments for GA [5, 6]. Hence, future clinical trials investigating treatments for GA will need to include tests of visual function.

The most common test of visual function in ophthalmology is high‐contrast best‐corrected visual acuity (BCVA) using a Snellen or ETDRS (Early Treatment Diabetic Retinopathy Study) letter chart. Whilst BCVA is a relatively reliable measure of many ophthalmic pathologies, it is not an early nor sensitive measure of GA severity. Due to the slow‐growing, parafoveal distribution of GA growth, both the fovea and BCVA are often unaffected until late in the disease process. Large areas of extrafoveal GA on FAF can cause minimal BCVA decline, whereas smaller lesions involving the fovea may produce disproportionate functional deficits. In addition, areas of retina spared of GA may not be large enough to ‘fit’ larger reading chart letters [7], confounding measurements. Once the fovea is affected by GA, BCVA usually deteriorates considerably and is permanently impacted, although this effect is delayed by a mean of 17 months [8]. Unlike other tests such as reading speed, BCVA does not account for testing time.

Ancillary testing of visual function, using methods such as low luminance visual acuity (LLVA), reading speed, contrast sensitivity, microperimetry, flicker perimetry, and dark adaptation, can provide a more holistic assessment of vision loss in GA [2, 9, 10, 11]. Whilst many visual function tests are not yet routinely applied in clinical practise due to logistical challenges, they play an increasingly important role in GA clinical trials [12] as demonstrating functional improvement is essential in achieving regulatory approval in some jurisdictions [5, 6]. It is therefore important for clinicians to better understand these modalities and their correlation with the more commonly used and familiar structural assessments. This review aims to evaluate and summarise tests of visual function and functional vision in the context of GA and compare them to structural tests, in particular FAF.

## 2 Materials and Methods

**FIGURE 1:**
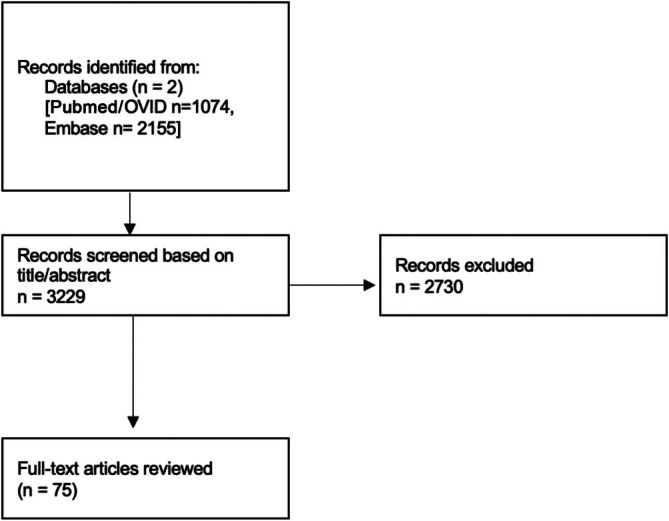
Literature search of functional tests for geographic atrophy.

A comprehensive literature search was carried out using Pubmed, Medline, and Embase over the course of January 2024 to March 2025. There were no limits on the year of publication. Articles were screened by the authors, and those deemed relevant for review were included (Figure 1). The search terms used included the key words ‘age‐related macular degeneration’, ‘geographic atrophy’, ‘visual function’, ‘low luminance visual acuity’, ‘reading speed’, ‘contrast sensitivity’, ‘microperimetry’, ‘flicker perimetry’, ‘dark adaptation’ with Boolean operators. Inclusion criteria were full‐text articles published in English regarding humans and adults aged 18 years and over. Types of articles included clinical trials, editorials, evaluation studies, guidelines, meta‐analyses, multi‐centre studies, observation studies, practise guidelines, randomised control trials, reviews, and systematic reviews. Articles were assessed by the authors (YL and ATF), and those deemed relevant to the aim of the present review were included.

## 3 Results

A comprehensive literature search yielded 3229 articles. 2730 articles were excluded following screening of the title or abstract. Out of the remaining articles with relevance to the topic, 75 full‐text articles were reviewed in detail (Figure 1).

### 3.1 Low Luminance Visual Acuity

**FIGURE 2:**
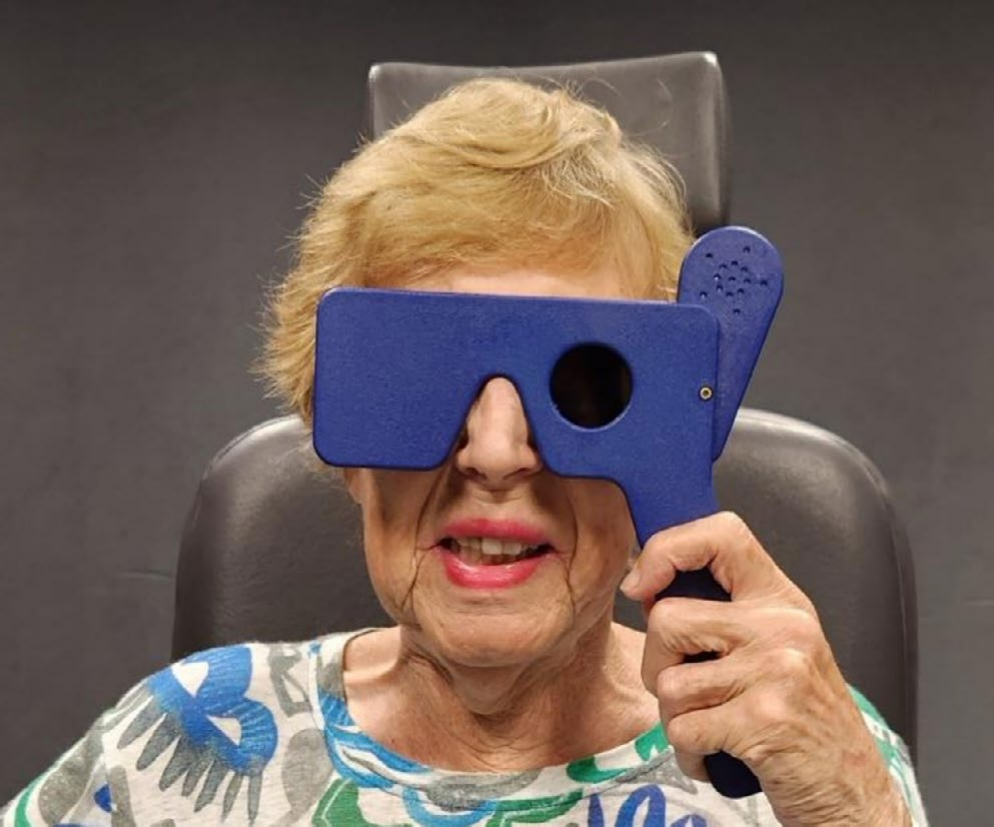
Testing low luminance visual acuity. An occluder with a 2.0‐log unit neutral density filter in front of the test eye is used.

Low Luminance Visual Acuity (LLVA) evaluates VA in dim, mesopic conditions. BCVA with an ETDRS chart is assessed at a luminance level of 100 times lower than room lighting, which can be achieved by using a 2.0‐log unit neutral density filter over the test eye (Figure 2) [13, 14]. Low‐luminance deficit (LLD) can then be calculated as the difference between LLVA and BCVA under standard conditions [13]. This is a sensitive and reproducible test in both the monitoring and prognostication of GA [13, 15].

The ability of photoreceptors to undertake dark adaptation underlies the physiological basis of LLVA, which is reduced in GA [9]. Another possible explanation behind reduced LLVA in GA is related to Müller cell injury in atrophic AMD, resulting in a reduction of light transmission to photoreceptors and subsequent decreased light sensitivity [16]. Eyes with extrafoveal GA generally exhibit a greater LLD compared to subfoveal GA [17], which is likely secondary to the high density of rods away from the fovea, as well as a poor photopic BCVA in eyes with foveal GA. Additionally, in foveal GA, as both BCVA and LLVA are reduced, the LLD is less likely to be large.

LLVA has been shown to achieve higher sensitivity in the detection of early reductions in retinal sensitivity compared to BCVA, particularly in extrafoveal GA [13]. LLD was also found to be greater in eyes with GA compared to those without [9, 18]. Patients with poor LLVA were more likely to develop subsequent vision loss, as tested on BCVA, at 2 years [13, 15]. However, the effectiveness of LLVA in the quantification of retinal function prior to the development of GA is variable. Some studies have found it to be a sensitive measure of retinal function in early and intermediate AMD [19, 20], whilst others did not find a difference in LLD in early AMD compared to controls [18, 21].

The reduction in LLVA in GA can be correlated with changes in structural imaging. Although better than BCVA, there is a positive but weak association between LLVA and GA lesion size on FAF, which is better with subfoveal lesions compared to extrafoveal lesions [17, 22]. Whilst the association between LLVA and FAF can be better defined, emerging evidence supports a link between LLVA and OCT biomarkers [23]. Signs of photoreceptor degeneration on OCT, particularly in the parafoveal region, have been shown to correlate with LLVA [23]. LLD has also been shown to correlate with a reduction in central macular choriocapillaris flow in eyes with intermediate and advanced AMD [24]. This correlation has offered further evidence that GA progression is correlated with changes in choroidal circulation. Using OCT‐angiography, Shen et al. [24] reported a positive correlation between LLD, outer retinal layer thickness, and choriocapillaris flow deficit.

### 3.2 Reading Speed

**FIGURE 3:**
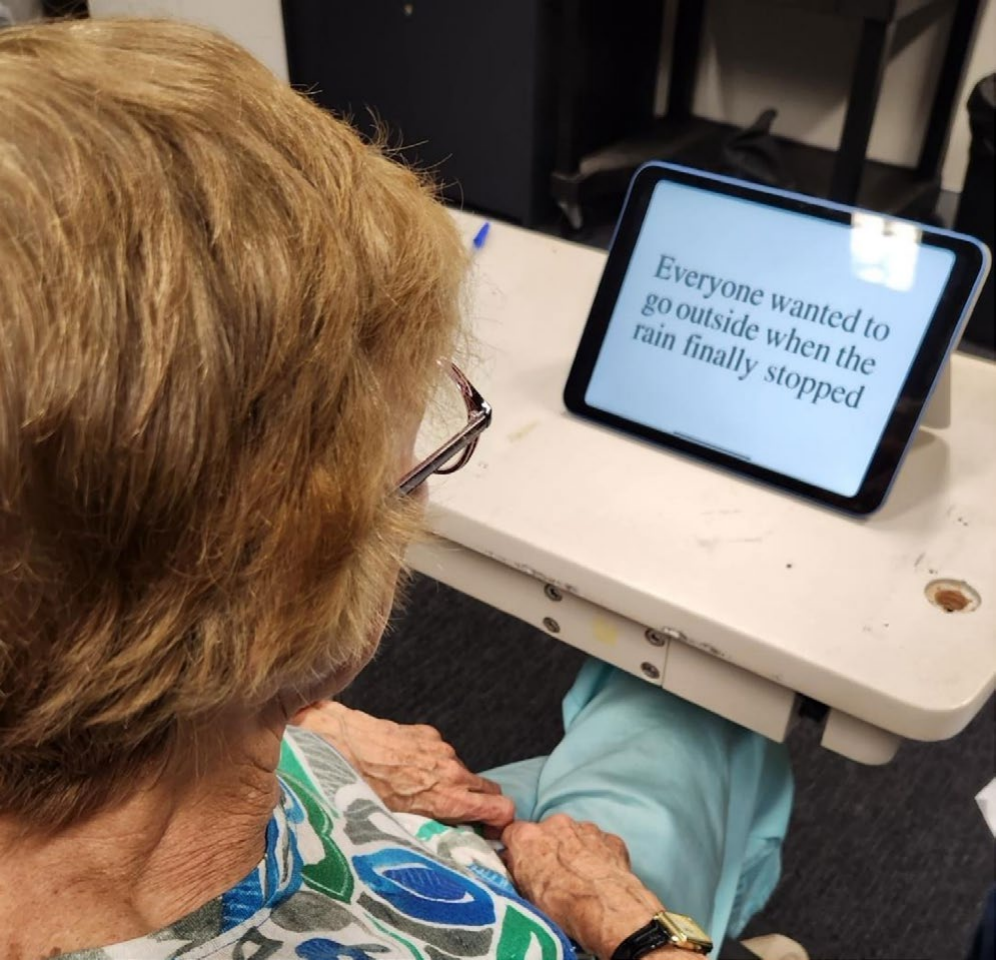
Minnesota low‐vision reading acuity chart assessed using a tablet.

Reading speed is another indicator of the extent of GA in patients with extrafoveal disease, where BCVA may be preserved [9]. As reading plays an important role in day‐to‐day function, assessment of reading speed can also indicate the potential hindrance of disease on ADLs and vision‐related quality of life. Various charts can be used to assess reading speed [25], such as the Minnesota low‐vision reading (MNREAD) acuity chart (Figure 3) and Radner chart, which are commonly used for patients with AMD [26]. Using the MNREAD acuity chart, Varma et al. [27] reported a better correlation between maximal reading speed with both GA lesion size and progression in comparison to BCVA. Similarly, Kunzel et al. [28] found that reading speed correlated with structural imaging biomarkers of GA as well as microperimetry.

A strong correlation between reading speed and GA size was found by Sunness et al. in 41 eyes with GA, but not with character size or BCVA [29]. In cases of extrafoveal GA, an increased reading speed was found to correlate with smaller character size [30]. Long‐term follow‐up of this study showed that this correlation persisted at five‐year follow‐up [31]. An analysis of 940 participants from the Chroma and Spectri trials showed a statistically significant but weak correlation between reading speed and GA lesion size on FAF and near‐infrared reflectance imaging [32]. Although this correlation was weak, it was superior to that between GA lesion size and BCVA [32]. The relationship between reading speed and GA size can possibly be explained by the presence and location of an eccentric preferred retinal locus (PRL) for fixation [31]. Eyes with a superior or right pattern PRL had a better reading rate, which is likely related to the direction of reading from left to right [31]. Based on this correlation, inferences regarding impairment in reading speed can be made from the location and size of GA on FAF. The weakness in correlation between GA lesion size and reading speed may be explained in part by character inversion (‘typoglycemia’), the differential importance of identifying first and last letters of a word for word recognition [33].

### 3.3 Contrast Sensitivity

**FIGURE 4:**
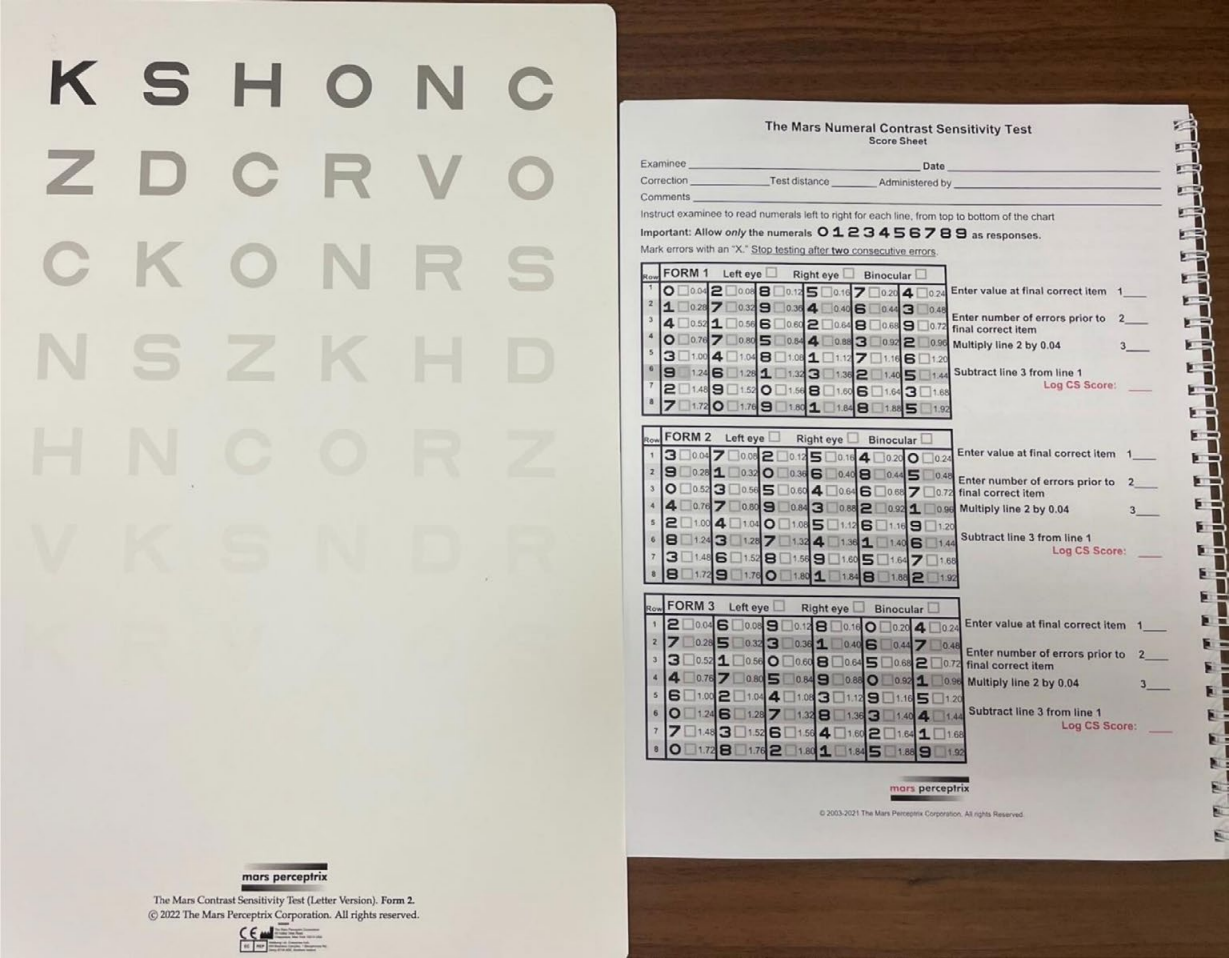
The Mars contrast sensitivity test.

Contrast sensitivity testing is widely adopted in the assessment of various macular pathologies, including GA [11]. Various techniques can be used to evaluate contrast sensitivity, such as the Pelli‐Robson Chart and Mars Letter Contrast Sensitivity Test (Figure 4). The Pelli‐Robson chart, usually tested at 1 m, utilises 16 sets of triplets of Snellen letters that fade in contrast by 0.15 log units. The Mars Contrast Sensitivity Test is conducted at a closer distance (50 cm) and allows for a more detailed measurement of contrast sensitivity, as each letter reduces by 0.04 log units. Whilst BCVA assesses the ability to resolve small details in high‐contrast settings, proficiency in contrast sensitivity is crucial in the performance of low‐contrast visual tasks such as facial recognition and most ADLs [34]. Contrast sensitivity can also serve as an additional surrogate marker of near vision‐related quality of life. In a study of 47 patients with advanced AMD, Roh et al. [35] reported a significant correlation between both contrast sensitivity and high contrast VA with vision‐related quality of life using the NEI VFQ‐25.

There is evidence to suggest a correlation between contrast sensitivity and structural changes on OCT and FAF. In a cohort of 205 eyes with intermediate AMD, eyes that progressed to GA were found to be associated with reduced contrast sensitivity at 1 cycle‐per‐degree, higher RPE volume in the inner ring, hyporeflective drusen cores, and subretinal drusen deposit [36]. Compared to BCVA, contrast sensitivity showed a stronger correlation with total GA lesion size and longitudinal growth in GA size in eyes with foveal GA [37]. Using Pelli Robson charts, Hoffmann et al. [22] found a significant correlation between contrast sensitivity with both GA size and the presence of foveal GA in eyes with neovascular AMD. Compared to BCVA and LLVA, contrast sensitivity had a stronger correlation with GA lesion size on FAF [22].

### 3.4 Microperimetry

Microperimetry assesses the macular visual field by mapping differential light sensitivity across a pre‐defined grid [38]. Light stimuli of varying intensity are used to determine the dimmest threshold for the perception of light. The main advantage of microperimetry over standard automated perimetry (SAP) is that the former allows eye‐tracking using a scanning laser ophthalmoscope, and the targets can be moved in real time to ensure better accuracy of repeat testing. Microperimetry is also an excellent method to determine fixation stability for patients with central vision loss, such as in patients with subfoveal GA.

Fixation stability is reduced with GA, as demonstrated by an increase in physiological micro‐saccades and a reduced ability to maintain focus on the central fixation cross during microperimetry testing. Patients with foveal GA often adopt eccentric fixation with a PRL outside of areas of scotoma, particularly if they have received targeted visual training to utilise these PRLs [31]. The use of a PRL in patients with a central scotoma is a beneficial compensatory mechanism, which is correlated with improved reading speed [39].

**FIGURE 5:**
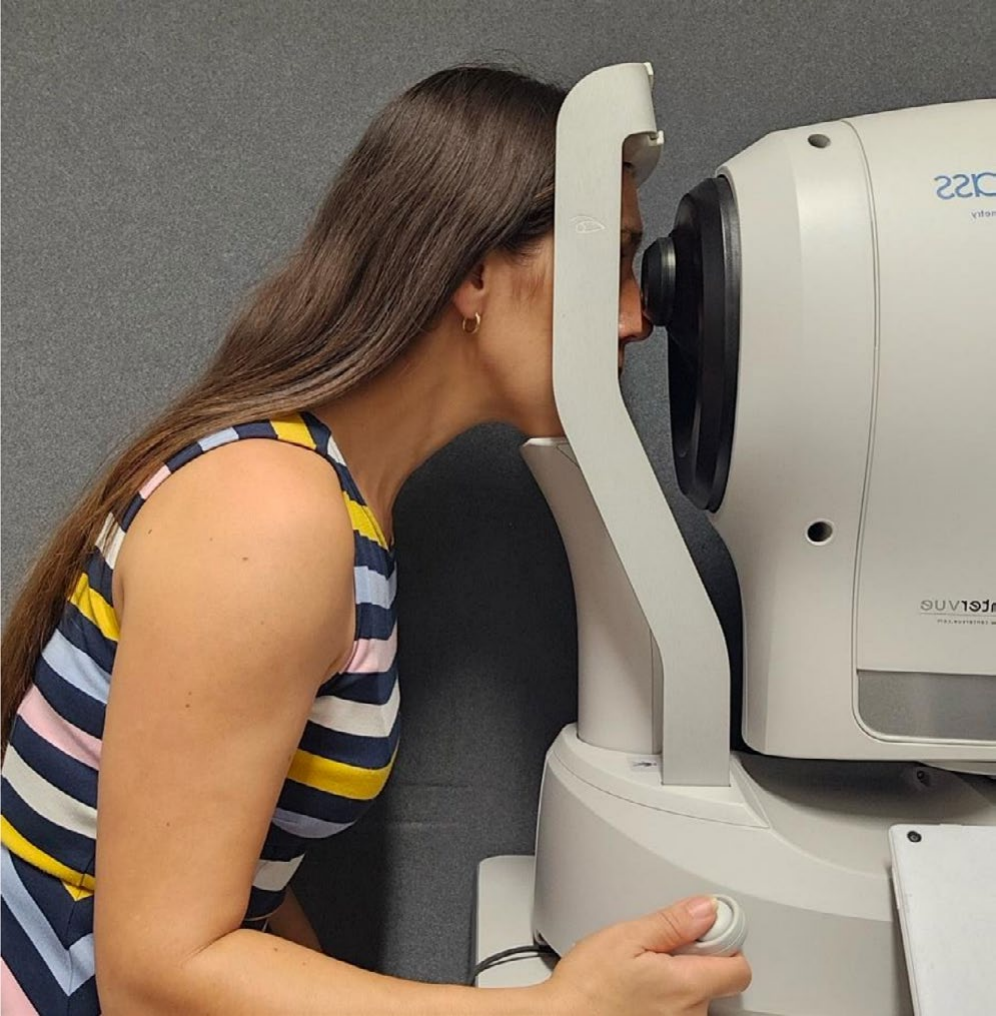
The COMPASS Microperimeter (CenterVue, Padova, Italy). The response button is held in the patient's right hand. The presence of the head rest helps to stabilise head motion to reduce movement artifact.

Various devices are commercially available for microperimetry, including Nidek MP‐1 and MP‐3 (Nidek Technologies, Gamagori, Japan), Optos Optical Coherence Tomography/Scanning Laser Ophthalmoscope (OCT/SLO, Optos Inc., Malborough, USA), iCare Macular Integrity Assessment (MAIA, CenterVue, Padova, Italy), and COMPASS (CenterVue, Padova, Italy, Figure 5) [40]. MP‐3 projects stimuli using a liquid crystal display, MAIA uses a combination of a light‐emitting diode and steering mirror, and scanning laser ophthalmoscopy is used in both Optos OCT/SLO and COMPASS [41, 42]. Test–retest studies on healthy eyes have demonstrated good repeatability of the above devices, with satisfactory inter‐device agreement when comparing MAIA with MP‐1 [43] and MP‐3 [41], and when comparing MP‐1 with Optos OCT/SLO [44]. However, analogous to devices used for biometry, interchangeability of devices for clinical trials and clinical practise is not recommended.

Decreases in microperimetric sensitivity usually precede BCVA and LLVA losses [18]. In addition to its use in the quantification of visual function in GA, abnormalities on microperimetry may also arise in areas of drusen, reticular pseudodrusen, and RPE changes in early and intermediate AMD [45, 46, 47]. One particular benefit of microperimetry over other tests is its ability to overlay structure with function, through the amalgamation of microperimetry maps with en‐face OCT to refine and define areas of disease progression, particularly in cases of nascent or perilesional GA [4, 40]. In addition to retinal sensitivity, fixation stability can be used to assess the progression of GA to absolute scotomata [38, 40].

The main challenge in the utilisation of microperimetry is patient performance, given the demographics of patients with AMD, who are often elderly with various other ocular and medical comorbidities. Testing results may be unreliable and incomplete in patients with cognitive decline, physical fatigue, or media opacity [40]. Testing duration of microperimetry takes at least 5 min per eye, where one study found the average time range between 12 and 13 min for patients with AMD [48]. This can be a disadvantage and confounding factor to its application in monitoring disease progression, as patient fatigue can impact result reliability. There is also a learning effect with microperimetry, and hence the first one or two tests are less reliable than future testing.

Many studies have evaluated the correlation between microperimetry and structural changes on FAF and OCT in both early and advanced AMD [4, 49, 50]. In the Chroma and Spectri trials of intravitreal lampalizumab for GA, microperimetry had the highest correlation with GA compared to BCVA, LLVA, reading speed, NEI VFQ‐25, or FRI Index [17, 51]. The binocular nature of the latter tests may underpin this finding, as the inclusion of the better‐seeing eye in testing may dilute the correlation [17]. The correlation between GA lesion size and microperimetry is less likely to be influenced by GA lesion location, as the degree of foveal involvement is more likely to affect metrics such as reading speed, NEI VFQ‐25, and FRI index [17]. Further analysis of microperimetry data from the Chroma and Spectri trials showed that the change in GA lesion size correlated best with the number of absolute scotomatous points, followed by perilesional (average sensitivity immediately adjacent to absolute scotomatous points) and responding sensitivity (average sensitivity of all non‐scotomatous points) [51]. Mean macular sensitivity had a weak correlation with GA lesion size, which was deemed likely secondary to the nature of its inclusion of all tested points [51]. The use of perilesional and responding sensitivity was proposed to be the most optimal metric for detecting subtle changes in retinal sensitivity with GA treatment, as they provide spatial information regarding the changes occurring at the crucial progression‐defining location [51].

Defect‐mapping microperimetry specifically evaluates deep visual sensitivity losses by presenting a suprathreshold stimulus a single time at each location, which maximises spatial density [4, 52]. Its sensitivity to lesions of varying sizes and sustained correlation with GA lesion progression over time was demonstrated in longitudinal studies over 2 years, where declines in visual sensitivity paralleled lesion growth for both lesions > 175 μm and lesions < 0.75‐disc areas [52, 53]. This method has been shown to exhibit a strong structure–function correlation with FAF whilst maintaining a testing time of around 5 min per eye, [4, 52] making it a promising tool for assessing treatment effect in GA treatment trials and supporting its logistical applicability to clinical practise [54].

The correlation between microperimetry and GA is most prominent at the junctional (‘transition’) zone, where an abrupt change in retinal sensitivity can be seen at the border just outside areas of complete atrophy [55, 56]. This pattern was consistently shown using defect‐mapping microperimetry, where stimulus sensitivity at the junctional zone was significantly reduced compared to anatomically distal locations [4, 54]. In 24 eyes with subfoveal and extrafoveal GA, a positive correlation was found between zones with absolute scotoma on microperimetry and hypoautofluorescence on FAF, which was superior to the correlation with choroidal hypertransmission on OCT [57].

The impact of various OCT imaging features on microperimetry performance has been explored by various studies [58, 59]. For example, when comparing the mean retinal sensitivity overlying various OCT structural changes, lesions such as atrophy and fibrosis scored the lowest, followed by intraretinal fluid and subretinal fluid, whilst pigment epithelial detachments (PEDs) had the least impact [58]. OCT biomarkers have variable correlation with microperimetry, where the features of choroidal signal hypertransmission ≥ 500 μm, complete RPE loss ≥ 250 μm, nascent GA ≥ 500 μm, external limiting membrane (ELM) disruption, and outer nuclear layer (ONL) thickness were independently found to correlate better with deep visual defects [60, 61]. In contrast, the relationship between scotoma density and the presence of complete RPE and outer retinal atrophy (cRORA) or incomplete RPE and outer retinal atrophy (iRORA) was less consistent, as not all cRORA lesions corresponded to increased scotoma density [60, 61]. In a study of 140 eyes with early, intermediate, and advanced AMD and 66 normal eyes, Tan et al. [58] found that the mean retinal sensitivity of perilesional and structurally normal areas was lower in patients with AMD than those without.

### 3.5 Flicker Perimetry

Flicker perimetry assesses retinal response to flickering perimetric stimuli. An increased metabolic demand is required for the retinal response to flicker stimuli, which is impaired in AMD due to the thickening of Bruch's membrane and a subsequent reduction in the metabolic supply to the retina [62]. Compared to normal eyes, eyes with drusen, PED, and GA exhibit a reduction in flicker perimetry sensitivity. Flicker perimetry offers higher sensitivity in detecting visual function decline in cases of early AMD, where it was shown to reveal a greater extent of retinal sensitivity loss compared to static perimetry [62, 63].

Flicker perimetry has also been shown to be a prognostic indicator in predicting the spatial and temporal progression to GA [63, 64]. In a longitudinal study of 127 eyes with AMD and 24 controls, the presence and rate of reduction in flicker sensitivity were shown to precede the clinical manifestation of both GA and neovascular AMD by several months [63]. As GA progresses, there is a reduction in sensitivity within areas of atrophy and a reduction in sensitivity in the junctional zone [63]. As flicker perimetry provides risk stratification for the progression of GA [63], further investigation into its ability to determine the direction of atrophy growth and the likelihood of developing subfoveal GA may help to select candidates for GA treatment.

Various commercially available automated perimeters possess the flicker perimetry function, such as the Heidelberg Edge Perimeter (HEP, Heidelberg Engineering, Heidelberg, Germany) [65], the Octopus 311 (Haag Streit, Koniz, Switzerland) [66], and the M700 automated perimeter (Medmont International Pty Ltd., Vermont, Victoria, Australia) [63, 64]. Although commonly used in glaucoma, the range of flicker perimetry devices specific to AMD is not well reported, which is in part related to the sparsity of published studies regarding their precision and reliability. Studies involving flicker perimetry in AMD predominantly utilise the M700 automated perimeter, which is comparable to the Humphrey Field Analyser in terms of scotoma localisation, central threshold, scotoma size, and testing time [67].

Some studies have investigated the correlation between flicker perimetry and structural imaging. Interestingly, Luu et al. [63] found that retinal sensitivity on flicker perimetry was reduced in seemingly structurally normal areas in eyes with GA. Importantly, these subclinical locations with reduced flicker sensitivity then progressed to GA throughout follow‐up at a higher rate, compared to eyes with stable flicker sensitivity in 127 patients with AMD and 24 controls [63]. This poses the promising role of perimetry in prognostication and predicting the onset of GA in eyes with AMD.

### 3.6 Dark Adaptation Time

Photoreceptor dark adaptation, which is the recovery of visual sensitivity in scotopic environments following exposure to photopic conditions, declines with age and can be impaired in a variety of retinal pathology [68]. Dark adaptation, occurring at luminance levels of 10–10^−6^ cd/m^2^, involves the recovery of light‐bleached rhodopsin through regenerating opsin, which is a relatively slower process that takes up to 20 min in cones to 60 min in rods [69]. Predominantly occurring in the photoreceptor outer segments, it is an intricate process involving the outer retinal layers, the RPE, and Bruch's membrane, which is orchestrated through pupil dilation, alterations in the phototransduction cascade, and various retinal neuronal interactions [69, 70]. Changes in the speed of dark adaptation portray the integrity of photoreceptor function, which can be a surrogate marker for visual function in various retinal conditions including AMD [70].

Dark adaptation is sensitive to impaired photoreceptor function, including cases of early AMD, often before structural changes are seen. Specifically, delayed rod‐mediated dark adaptation (RMDA) is typically seen in eyes with AMD, whereas cone‐mediated dark adaptation is generally unaffected [71]. A longitudinal study over 3 years showed that eyes with seemingly structurally normal maculae, but delayed RMDA, were 2 times more likely to develop AMD compared to eyes with normal RMDA [72]. In the Alabama Study on Early Age‐related Macular Degeneration studies, Owsley et al. [73] demonstrated a reduction in RMDA in eyes with early and intermediate AMD, with graduated deficiency when comparing early to intermediate AMD. Notably, eyes with reticular pseudodrusen (RPD), also known as subretinal drusenoid deposits (SDD), exhibited a longer rod intercept time than those without [73, 74], highlighting its role as a biomarker of poor retinal health. In eyes with early or intermediate AMD, location‐targeted RMDA was found to be slower in locations with SDD at 5° and 12° from the fovea [75]. However, despite its sensitivity as the first measurable functional deficit, it is important to note the long testing time involved in assessing dark adaptation, with at least 20 min per eye, considering the amount of time required to physiologically undertake dark adaptation. This significantly limits the feasibility of its application in clinical practise.

**FIGURE 6:**
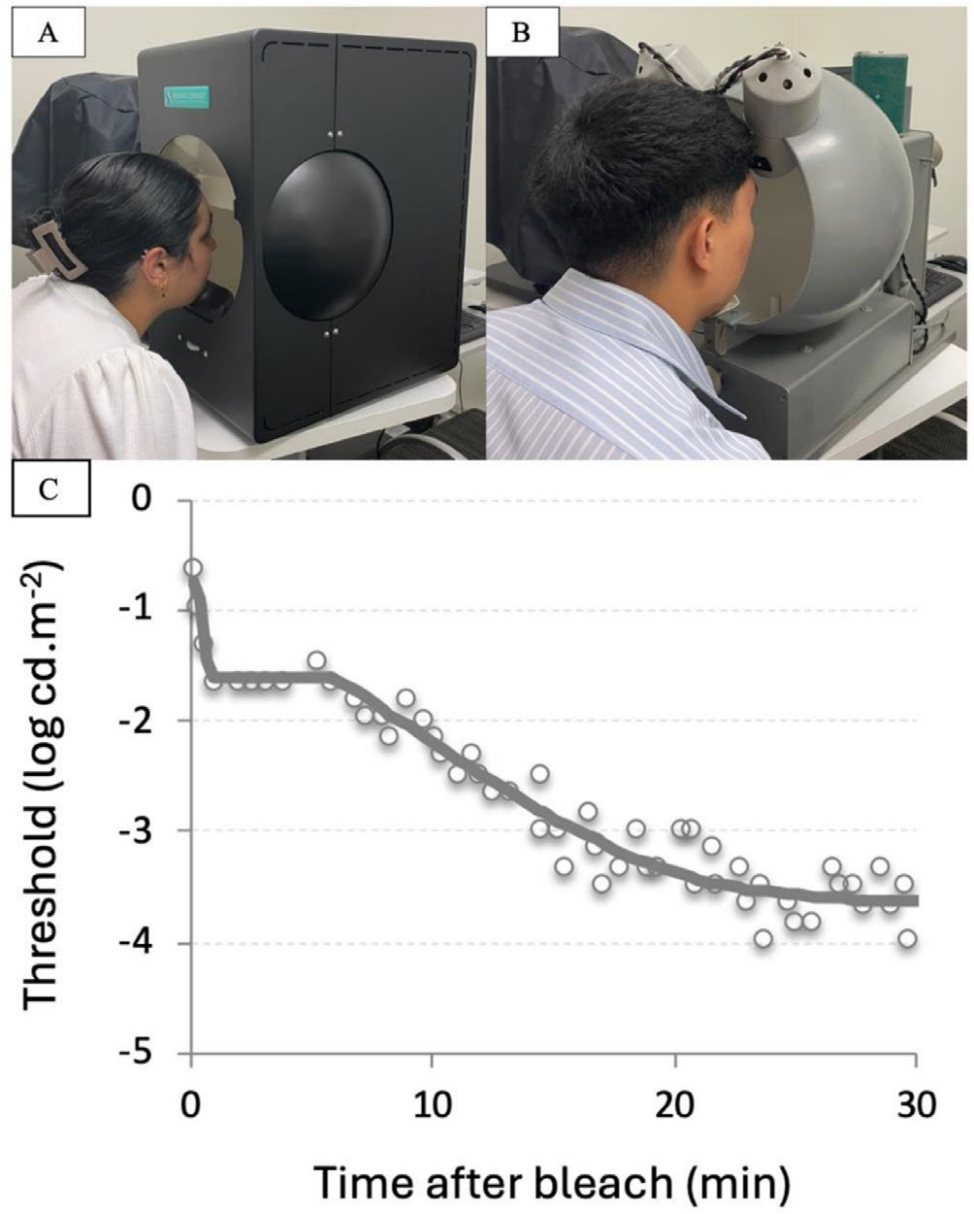
Dark adaptometers. The Roland (A) and Goldmann‐Weekers dark adaptometers (B). Tests are generally conducted in dim conditions. A dark adaptation graph from a normal patient is shown in (C).

Commercially available devices have been validated for the evaluation of dark adaptation (Figure 6), including the AdaptDx (Apeliotus Technologies, Atlanta, GA, USA), Roland Consultant Dark Adaptometer (Roland Consultant GmbH, Germany, Figure 6A), and the Goldmann‐Weekers Dark Adaptometer (Haag Streit, Koniz, Switzerland, Figure 6B) [76]. Medmont Dark Adapted Chromatic Perimeter (Medmont Pty Ltd., Melbourne, Victoria, Australia) performs additional functions, including the ability to measure dark‐adapted perimetry in various locations [77]. AdaptDx is the most commonly reported device, which measures rod intercept time, the amount of time required for visual sensitivity at a single retinal location to return to a stimulus intensity of 3‐log units lower than the initial threshold, following photoreceptor bleaching with a bright flash of up to 5.8 × 10^4^ cd‐s/m^2^ intensity [76].

Both cross‐sectional and longitudinal studies have shown a significant correlation between AMD severity and dark adaptation impairment [78, 79]. Additionally, changes in dark adaptation may occur despite stability in BCVA, suggesting better sensitivity in the assessment of visual function loss in patients with GA [78, 80]. The presence of RPD is hypothesised to compromise dark adaptation through its impact on Vitamin A transport across the Bruch's‐RPE complex and the subsequent impairment in the visual cycle [78]. Structural OCT changes on OCT, such as hyperreflective foci, incomplete retinal pigment epithelial and outer retinal atrophy (iRORA), and drusenoid PEDs, also correlate with an impairment in dark adaptation [81].

Dark adaptation has a promising role in predicting the likelihood of developing advanced AMD due to the increased risk of developing GA and neovascular AMD in patients with RPD [82]. However, its role in monitoring the progression of GA and sensitivity in correlation with structural changes and in comparison to microperimetry, however, is still unknown. Longitudinal studies of dark adaptation changes in various lesion sizes and locations would be helpful to better define and standardise its role in GA monitoring. As the tedious nature of the test reduces its clinical feasibility, correlation with structural markers on OCT can be a helpful tool in predicting the development and progression of GA.

### 3.7 Patient‐Reported Outcomes

Patient‐reported outcomes can complement visual function testing, as it helps to determine functional vision and the extent of disability inflicted by GA. GA is known to cause significant functional and psychosocial burden, where patients with subfoveal GA struggle with facial recognition, driving and navigation, and parafoveal GA causing significant issues with reading and complex visual scenes [83]. GA is also known to lead to a higher incidence of mental health issues, including anxiety and depression [84]. As a result, there are substantial downstream effects on loss of hobbies, reduced socialisation, and loss of independence.

Despite the significant impact of GA on vision‐related quality of life and daily functioning, functional outcomes beyond VA, such as driving, facial recognition, and night vision, are inconsistently assessed and documented in a clinical setting [85]. Patient questionnaires can directly assess the impact of GA on ADLs and serve as an approximation of perceived functional vision, as logistical difficulties limit the ability to assess these outcomes formally. Outcomes from the questionnaires can be extrapolated to approximate patients' ability to perform ADLs. However, although driving ability can be estimated using BCVA and visual field defects according to regulatory criteria, this may not reflect the patient's actual comfort or confidence with driving.

Frequently used questionnaires to assess the impact of vision loss in patients with GA include the National Eye Institute Visual Functioning Questionnaire‐25 (NEI VFQ‐25), Functional Reading Independence Index (FRI), and Impact of Vision Impairment (IVI) questionnaire [86, 87, 88, 89]. It is important to note that as the questions assess visual function with binocular vision, they may underestimate visual impairment in patients with unilateral GA.

The NEI VFQ‐25 is a validated and reliable questionnaire that is widely used in both clinical practise and clinical trials [90, 91]. The NEI VFQ‐25 evaluates the impact of vision loss on ADLs, social functioning, and mental health secondary to vision difficulties [86]. A score between 0 and 100 is generated, where a higher score indicates better visual function. Results of the NEI VFQ‐25 have been shown to correlate with BCVA, reading speed, and contrast sensitivity [86]. The NEI VFQ‐25 has also been shown to correlate with LLVA [92]. Kunzel et al. [92] assessed the relationship between NEI VFQ‐25, LLVA, and GA location and found LLVA to be the most important variable in determining vision‐related quality of life, which was emphasised in patients with extrafoveal GA. Outcomes from the NEI VFQ‐25 suggest a profound level of impairment with functional vision and ADLs in patients with GA [33, 34].

The FRI index assesses the overall level of independence in performing functional reading activities through evaluating the patient's level of independence with activities such as reading labels, reading bills, reading medicine labels, and reading print on a screen [87]. Significant correlations have been found between the FRI index and GA lesion, NEI VFQ 25 score, and maximum MNREAD acuity chart reading speed [87].

The IVI is a questionnaire that assesses the impact of vision impairment on the ADLs based on five domains, including mobility, personal cares, social interactions, leisure and work‐life, and emotional response to reduced vision [88, 89]. Multiple studies have validated the reliability of the IVI, in terms of its validity, repeatability, and correlation with VA [89, 93]. The IVI can serve as a bridge between vision function measurements, functional vision, and vision‐related quality of life [94]. In a study of patients with early, intermediate, and late AMD, LLVA and contrast sensitivity were found to correlate with vision‐related quality of life as assessed using IVI [94].

## 4 Discussion

The various visual function tests outlined in this review assess distinct facets of vision loss that are caused by GA, with varying degrees of correlation with structural assessment using FAF. Visual function tests differ in utility depending on disease stage, extent, and location [95]. BCVA is helpful for foveal‐involving GA. In unifocal or multifocal extrafoveal GA, microperimetry is particularly useful as tests that depend on foveal function, such as BCVA and LLVA, may not detect changes adequately [95]. Reading speed may be influenced by GA lesion location due to differences in PRL, which may be particularly informative for assessing extrafoveal multifocal lesions [95].

### 4.1 Structure–Function Correlation

Each of the tests of visual function has its advantages and disadvantages (Table 1). LLVA, reading speed, and contrast sensitivity are relatively inexpensive and have a short testing time. All three have been shown to correlate weakly with GA lesion size on FAF, although with a better correlation compared to BCVA. Contrast sensitivity has a relatively stronger correlation than LLVA. Functional vision is strongly influenced by contrast sensitivity, given its crucial role in enabling various activities of daily living. The correlation between FAF and visual function tests is largely determined by GA lesion location in relation to the fovea. LLVA, reading speed, and contrast sensitivity are variants of BCVA that rely on fovea function, which do not correlate well with extrafoveal GA as visualised by FAF. In contrast, microperimetry and flicker perimetry provide location‐specific information, which may correlate better with FAF‐detectable GA irrespective of its relationship to the fovea.

**TABLE 1:** A comparison of currently available tools for visual function testing.

	Device examples	Advantages	Disadvantages
1. Low luminance VA	VA with a 2.0‐log unit neutral density filter	Inexpensive	May be difficult to standardise lighting conditions with varying luminance levels
		Short testing time	
2. Reading speed	Minnesota low‐vision reading (MNREAD) acuity chart	Inexpensive	Difficult to use in non‐English speaking patients
	Radner chart	Short testing time	Can be confounded by medical comorbidities
			Subjective
3. Contrast sensitivity	Pelli‐Robson Chart	Inexpensive	Difficult to use in non‐English speaking patients
	Mars Letter Contrast Sensitivity Test	Short testing time	Subjective
4. Microperimetry	Nidek MP‐1 and MP‐3 (Nidek Technologies, Gamagori, Japan)	Precise	Patient performance dependent
	Optos Optical Coherence Tomography/Scanning Laser Ophthalmoscope (OCT/SLO, Optos)	Direct correlation between structure and function	Time‐consuming
	Macular Integrity Assessment (MAIA, CenterVue, Padova, Italy)	Changes can precede structural abnormalities	Device may not be available in all clinics
	Compass (CenterVue, Padova, Italy)		Difficulties with use in non‐ambulatory patients
			Some devices have ceiling effects, where patients with better retinal sensitivity cannot be accurately assessed
5. Flicker perimetry	Heidelberg Edge Perimeter (Heidelberg Engineering, Heidelberg, Germany)	Precise	Patient performance dependent
	Octopus 311 (Haag Streit, Koniz, Switzerland)	Changes can precede structural abnormalities	Time‐consuming
			Device may not be available in all clinics
	M700 automated perimeter (Medmont International Pty Ltd., Vermont, Victoria, Australia)	Role in prediction and prognostication	Difficulties with use in non‐ambulatory patients
6. Dark adaptation time	AdaptDx and AdaptRx (Apeliotus Technologies, Atlanta, GA, USA)	Precise	Time‐consuming (> 1 h to complete for both eyes)
	Medmont Dark Adapted Chromatic Perimeter (Medmont Pty Ltd., Melbourne, Victoria, Australia)	Objective	Device not available in most ophthalmology and optometry clinics
	Roland Consultant Dark Adaptometer (Roland Consultant GmbH, Germany)	Changes can precede structural abnormalities	
7. Patient reported outcomes	National Eye Institute Visual Functioning Questionnaire‐25	Patient‐reported	Subjective
	Functional Reading Independence Index	Key clinical endpoint considered by medicine regulatory bodies	Difficult to use in non‐English speaking patients
	Impact of Vision Impairment		

Tests such as microperimetry, flicker perimetry, and dark adaptation showcase a different dimension of GA by generating quantitative plots that reflect the function of photoreceptors and retinal cells. Microperimetry has the strongest correlation with GA lesion size on FAF and also has the advantage of being able to overlay structure and function. In patients with GA, it correlates better with FAF than OCT. Flicker perimetry may be able to identify subclinical areas of GA and be useful for prognostication. Dark adaptation correlates with OCT structural changes of GA and is useful in early and intermediate AMD, but its role in GA needs to be better defined. The main disadvantage with these three investigations is their limited availability and time‐consuming nature of testing, ranging up to 13 min per eye with microperimetry and 20 min per eye with dark adaptation. The use of targeted, high‐density microperimetry and defect‐mapping microperimetry may lower the time required for testing and improve repeatability [4, 96]. The development of a faster screening algorithm with modifications to factors such as stimulus intensity, number of stimuli required, and the exclusion of false negatives and fixation testing, analogous to the Swedish Interactive Threshold Algorithm (SITA)‐fast and SITA‐faster strategy used in glaucoma, may help to reduce testing time and improve the clinical applicability of microperimetry.

Patient‐reported outcomes with the NEI VFQ‐25, FRI index, and IVI questionnaire approximate functional vision but may be subjective and limited by patient cognitive abilities. Administering the questionnaires at home prior to the clinic visit can improve their applicability and feasibility by mitigating time constraints in clinical practise.

### 4.2 Applications of Visual Function Tests

Understanding the characteristics of visual function tests in GA is highly relevant in the emerging uptake of GA treatments, particularly complement factor inhibitors. Intravitreal complement factor inhibitors, such as pegcetacoplan (Syfovre), have recently received regulatory approval by the US Food and Drug Administration (FDA) in February 2023 and the Australian Therapeutic Goods Administration (TGA) [97] in January 2025 for the treatment of GA [98]. Similarly, avacincaptad pegol (IZERVAY) was approved by the FDA [99] in August 2023 and the TGA [100] in October 2025. In September 2025, Japan also announced conditional approval for IZERVAY [101]. For pegcetacoplan, this was based on evidence from the pivotal phase 3 clinical trials OAKS & DERBY showing a reduction in the rate of growth of GA lesion size based on FAF [102, 103]. Structural benefit was taken as a surrogate marker of functional benefit, based on long‐term natural history data showing growth of GA can affect fixation patterns and reading rates [29, 31, 104]. These findings are supported by a Delphi study of international AMD experts, in which a consensus was achieved on the use of end‐stage atrophy detected by structural testing modalities that align with functional changes as a clinical endpoint for treatment trials [105]. In contrast, the European Medicines Agency (EMA) refused marketing approval of intravitreal pegcetacoplan for GA in June and September 2024 [5]. Similarly, the Medicines and Healthcare Products Regulatory Agency (MHRA) in the United Kingdom rejected the use of Syfovre [6]. This decision was based on a lack of prospective data showing evidence of benefit in visual function or functional vision, as the regulators did not feel there was sufficient evidence to show that decreased growth of GA lesions on FAF directly correlated to real‐world benefit for patients [5].

Approval of GA treatments by regulatory bodies such as the EMA will either require statistically significant benefits to be shown in pre‐specified tests of visual function in clinical trials or the emergence of stronger evidence supporting the correlation between structure and function. The temporal relationship between structural change and functional improvement also requires consideration. Although changes in dark adaptation and flicker perimetry precede structural changes [63, 73], functional benefit following a slowing of structural deterioration may not be instantaneous, necessitating the need for long‐term outcomes.

### 4.3 Discrepancies in Structure–Function Correlation

Potential reasons behind the lack of functional improvement despite GA growth in patients treated with complement inhibitors have been postulated previously [106, 107]. The tests used may be too blunt to identify changes of a slowly progressive disease within the short time frame of a clinical trial. The GALE open‐label extension study of pegcetacoplan for GA was the first time a visual functional benefit (number of scotomatous points on microperimetry) had been seen in an approved therapy for this disease. However, statistical significance was first seen only at the 36‐month time point [12]. Confounding factors such as lesion phenotype and the impact of fellow eye status may influence functional outcomes. The impact of other unknown factors that affect the structure–function interplay, such as genetics and pathogenic pathways outside the complement cascade [107, 108], may also alter changes in visual function.

Given GA growth and functional decline can be differentially influenced by lesion size, location, and focality [106], analyses of treatment response should be performed in a targeted manner rather than a one‐size‐fits‐all model. For example, a rapidly growing extrafoveal lesion may have less impact on visual function than a slowly expanding subfoveal lesion. Similarly, the degree of improvement in visual function is also dependent on the test of choice, where slowing lesion expansion in a subfoveal lesion may be more apparent on microperimetry than tests like patient‐reported outcomes and reading speed. This raises the need to design trials and post hoc analyses that target specific eye and patient cohorts [106]. Ideally this should utilise a range of visual function tests and include GA phenotypes that are most likely to benefit functionally from treatment. The OAKS, DERBY, and GALE studies have demonstrated that pegcetocoplan can effectively reduce the involvement of central scotomatous points on microperimetry in post hoc analysis [12]. These tests will need to be included prospectively in future clinical trials, using functional tests that are carefully matched to the GA phenotype, maximising their sensitivity in detecting meaningful changes.

### 4.4 Improving Structure–Function Correlation—The Search for Structural Biomarkers

Despite advances in our understanding of the structure–function relationship in GA, the exact functional impact of structural changes on multimodal imaging still requires further validation. Artificial intelligence (AI) and deep learning algorithms may hold the key to understanding and strengthening the association between structural changes and visual function [109, 110]. This includes correlation between OCT and microperimetry [110] or VA [111]. Using a deep‐learning algorithm, 3D OCT scans have been correlated with corresponding microperimetry examinations in patients with healthy maculae, early to intermediate AMD, neovascular AMD, and GA to generate a method of predicting retinal sensitivity based on various OCT biomarkers [110]. Using microperimetry, OCT, FAF, and near infrared reflectance imaging, Ansari et al. [112] found that the random forest model was reliable in determining features most predictive of retinal sensitivity loss, including thickness of the choroid, RPE‐Bruch's membrane complex, ellipsoid zone, ELM, and ONL. Also using random forest models, Pfau et al. [113] found that ONL thickness was the strongest predictor of retinal sensitivity and was able to generate inferred sensitivity maps that matched expected field deficits. Beyond the spatial borders of GA lesions, macula‐wide ONL thickness was found to be associated with GA progression [114], which could be considered a surrogate endpoint with the assistance of AI algorithms. The integration of structural data with functional outcomes through AI‐assisted algorithms may help to identify which structural biomarkers can most reliably predict decline in certain GA phenotypes. Establishing structural biomarkers is of particular importance in low‐resource settings, rural and remote practises, and high‐volume centres where visual function tests cannot be routinely performed.

## 5 Conclusions

Although structural tests for GA, such as FAF and OCT, are objective and have relatively simple acquisition, they are only a surrogate measure for visual function. BCVA is a poor metric for assessing function in GA. Of the other tests for visual function, LLVA, reading speed, and contrast sensitivity are the most accessible due to their cheaper cost and shorter testing time, but they can be subjective, difficult to standardise, or perform in patients of non‐English speaking backgrounds. They all show weak correlation with GA lesion size on FAF, although they have better correlation than BCVA. Contrast sensitivity has a better correlation than LLVA.

Microperimetry has the strongest evidence of correlation with GA lesion size on FAF. It also has the advantage of overlaying structure and function, particularly with the use of dark adaptation perimetry. Flicker perimetry shows promise in identifying subclinical areas of GA. Dark adaptation correlates with GA identified by OCT. These tests are limited by availability, testing time, and ceiling effect, such that they are impractical to implement in routine clinical practise. Unlike tests of visual function, patient‐reported outcomes attempt to test functional vision and are the closest to ‘real‐world’ assessment but are subjective.

A range of targeted, prespecified endpoints of visual function testing should be included in future clinical trials for treatments of GA, which should be matched to the GA phenotype and disease stage. In order to demonstrate functional benefit over a reasonable follow‐up period, studies should focus on GA lesion phenotypes that are known to progress rapidly. This is critical in jurisdictions where proof of functional benefit is required for regulatory approval of treatments for GA. Until the correlation between FAF and functional tests is more concrete, it may be useful to consider utilising functional tests in monitoring patients who are commenced on GA treatments in clinical practise. Tests such as LLVA, reading speed, and contrast sensitivity are relatively simple to implement in clinical practise, although their correlation with FAF is not as robust as microperimetry. The feasibility of implementing microperimetry in the real world may be variable between practises. Using a limited subset of testing points with emphasis on changes at the junctional zone using specific modalities, such as defect mapping microperimetry, can be considered to improve the logistical practicality of its implementation.

## Funding

The authors have nothing to report.

## Disclosure

Adrian T. Fung receives financial support from the following relevant companies: Roche (consultant, speaker fees, advisory board, research), Bayer (speaker fees, advisory board, research, travel), Novartis (speaker fees, travel), Apellis (advisory board), Alcon (speaker fees, advisory board), Allergan (speaker fees, travel, research), Ionis Pharmaceuticals (research), Astellis (advisory board), Samsara Vision (advisory board), AbbVie (lecture honoraria, travel research), Ophthea (equity), Macuject (equity), Biojiva (research). Lauren N. Ayton is supported by an NHMRC Investigator grant (GNT#1195713) and is a consultant to Janssen, PYC Therapeutics, Kiora Pharmaceuticals, Astellas, and VesperBio Therapeutics. The Centre for Eye Research Australia receives support from the Victorian State Government through its Operational Infrastructure Support Programme. No funding was received for this work. The authors warrant that this paper is original and has not been in part or in whole published in another journal.

## Conflicts of Interest

The authors declare no conflicts of interest.

## Data Availability

Data sharing not applicable to this article as no datasets were generated or analysed during the current study.
